# No Evidence That Nest Cover Shapes Incubation Schedules in a Mediterranean Shorebird Population

**DOI:** 10.1002/ece3.74225

**Published:** 2026-08-20

**Authors:** Hela Boughdiri, Grant C. McDonald, Tamás Székely, András Kosztolányi

**Affiliations:** ^1^ Department of Evolutionary Zoology and Human Biology University of Debrecen Debrecen Hungary; ^2^ Department of Zoology University of Veterinary Medicine Budapest Budapest Hungary; ^3^ Milner Centre for Evolution, Department of Life Sciences University of Bath Bath UK

**Keywords:** incubation behaviour, Kentish Plover, Mediterranean climate, nest cover, parental care, shorebirds

## Abstract

Nest site selection is a key reproductive decision in ground‐nesting birds, given its direct consequences for breeding success and survival of the parent(s). Studies in extreme hot environments show that cover (e.g., vegetation, rocks) that provides shade at the nest can strongly shape parental incubation behaviour. Understanding the generality of these effects across differing environments, including less extreme climates, is an important goal in predicting the behavioural and reproductive impacts of rising global temperatures. Here we investigated how nest cover influences biparental incubation effort and sex‐specific investment in a wild population of Kentish Plovers breeding in a Mediterranean environment. We used 24‐h video recordings from 24 nests with variable nest cover that ranged from fully open (0% coverage) to complete coverage (100%). Contrary to previous work that indicates less covered nests require more parental effort to shield embryos from thermal extremes, we found that nest cover was not associated with total incubation, mean off‐nest duration, or male incubation share. However, we detected a non‐significant trend for a higher frequency of off‐nest periods in more covered nests. Our results suggest that nest cover is not a strong modulator of incubation behaviour in our population, suggesting that in some circumstances nest cover may primarily impact aspects of incubation other than thermoregulation, such as protection from visual predators, wind exposure or precipitation. Despite no observed difference in incubation patterns, we cannot preclude the possibility that parents nesting in low cover sites are exposed to greater heat stress, but such heat stress and physiological costs are not reflected in changes in incubation. We propose comparative studies of biparental ground‐nesting species across climatic gradients to identify the adaptations associated with breeding in hot climates that include behaviour, heat tolerance and nest cover.

## Graphical abstract

We investigated how nest cover is associated to incubation behaviour in a Mediterranean biparental population of Kentish Plovers using 24‐h video recordings from 24 nests with variable nest cover that ranged from fully open (0% coverage) to complete coverage (100%). Contrary to our expectations, nest cover was not associated with overall incubation effort or sex‐specific investment, although we detected a weak positive trend towards more frequent off‐nest periods in covered nests. Our results indicate that nest cover may play a limited role in shaping incubation behaviour in moderate climates.

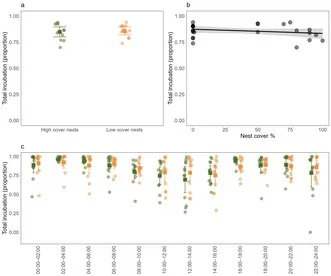

## 1 Introduction

Deciding where to nest is a complex process for nesting animals, as it has direct consequences on the individuals' breeding success and survival (Refsnider and Janzen 2010; Refsnider 2016; Lehtonen et al. 2023). This process is influenced by several factors including habitat characteristics, climatic conditions, predation pressure, parental condition and prior breeding experience (Wiebe and Martin 1998; Clark and Shutler 1999; Hoi et al. 2012; Mainwaring et al. 2017). In birds, incubation is among the most energetically costly stages of reproduction, as parents need to maintain their eggs within a specific thermal range for an extended period of time to ensure embryonic development, often at the expense of their own foraging opportunities (Williams 1996; DuRant et al. 2013). Parents should therefore nest in locations that shield eggs from solar exposure and temperature extremes, and thus minimise incubation effort and maximise parental incubation efficiency (Carroll et al. 2018; DuRant et al. 2019).

Nest cover is one such nest site parameter that may influence nest location selection and incubation behaviour (Götmark et al. 1995; Davis 2005; Mainwaring et al. 2014; Fogarty et al. 2017). Many shorebirds nest under the cover of vegetation, and to understand the variation in nest cover within and across species, it is important to uncover the costs and benefits of nesting in the open vs. in cover (Bulla et al. 2016). Open nests without vegetation cover may face a high overheating risk due to intense solar exposure, particularly in hot environments and thus may demand greater parental effort for the eggs' thermoregulation (AlRashidi et al. 2011; AlRashidi and Shobrak 2015, 2025). Covered nests with high cover can mitigate heat stress via shading parents and eggs, thus potentially reduce parental thermoregulation effort (Amat and Masero 2004a; Kim and Monaghan 2005; AlRashidi et al. 2011). The relationship between nest cover and incubation behaviour in biparental bird species has been studied both in hot to extremely hot systems. For instance, a study by Amat and Masero (2004a) on Kentish Plover in Spain (ground temperature may exceed 50°C) revealed that open nesting parents expressed thermoregulatory behaviours (such as panting or holding wings, shading the nest) to mitigate heat stress affecting both parents and eggs; however, parents at covered nests did not show such behaviours. In extremely hot systems such as the United Arab Emirates or Saudi Arabia (ground surface temperature often exceeds 50°C at midday), temperatures strongly influence incubation and parental cooperation (AlRashidi et al. 2010, 2011). Moreover, studies revealed that open nesting parents showed higher nest attendance and a higher number of changeovers between parents compared to covered nests, to maintain clutch temperature within a viable temperature range (AlRashidi et al. 2011). Another study by Clauser and McRae (2017) conducted on open nesting King Rails ( *Rallus elegans* ) revealed that parents had more frequent but shorter recesses in hotter conditions to cool themselves sufficiently.

While nest cover may have several benefits in terms of thermal regimes, there are also likely costs if nest cover hinders a parent's vision and therefore limits its ability to detect predators and escape rapidly (Albrecht and Klvaňa 2004; Gómez‐Serrano and López‐López 2014; Lomas et al. 2014; Dorsey et al. 2025). As a consequence, nest cover may influence the parents' antipredator behaviour, thereby affecting their incubation rhythms. For instance, parents nesting in sites with limited visibility may experience a larger information deficit and, as a consequence, react strongly to predator cues by leaving their nests more frequently to assess threats, thereby disrupting incubation more often than parents in more open sites (Schneider and Griesser 2013).

Understanding, the generality of these nest cover effects on avian incubation across differing environments is an important goal in predicting the behavioural and reproductive consequences of rising global temperatures (Mainwaring et al. 2017; DuRant et al. 2019; Székely et al. 2024). Despite valuable insights of nest cover studies into how ground‐nesting birds cope with thermal challenges, several gaps remain. First, the costs and benefits of nest cover, as well as its influence on incubation behaviour, may vary depending on local environmental conditions (Mainwaring et al. 2017). For instance, strategies identified in extremely hot environments (i.e., desert regions) may not apply to populations breeding in hot, yet less extreme habitats (Jin et al. 2025). Second, previous studies have often tested the relationship between incubation effort and nest cover as a dichotomous variable rather than a continuous variable, perhaps limiting the ability to detect gradual changes in incubation behaviour across the full range of nest cover (Amat and Masero 2004a; AlRashidi et al. 2011). Third, while nest cover may mediate parent cooperation over care during incubation under some conditions (Amat and Masero 2004a; AlRashidi et al. 2011), it remains unclear how nest cover may mediate the share of incubation effort between males and females in different thermal systems.

In this study we investigated the role of different nest cover strategies in shaping parental incubation in a ground‐nesting bird, the Kentish Plover (Anarhynchus *alexandrinus*, previously classified as *Charadrius alexandrinus* ; Černý and Natale 2022), a biparentally incubating species known for its flexible nesting and mating strategies (Lessells 1984; Amat et al. 1999; Székely et al. 2006; AlRashidi et al. 2011; Gómez‐Serrano and López‐López 2014). Kentish Plover is a sexually dimorphic species, with males demonstrating more elaborate plumage than females (Argüelles‐Ticó et al. 2016). The species breeds in a wide range of thermal environments across Europe, Asia and Africa (Schulz and Stock 1993; Amat and Masero 2004b; Hanane 2011; McDonald et al. 2022, 2025; Ding et al. 2023). Before nesting, male Kentish Plovers visit multiple potential sites and make multiple shallow scrapes, with the female closely following him and occasionally sitting in scrapes and contributing to scraping (Székely et al. 1999; Lendvai et al. 2004). Both Kentish Plover parents cooperate to incubate their clutch of eggs for approximately 25 days (Székely et al. 1994) with females primarily incubating during daylight hours and males primarily incubating in the evening (Amat and Masero 2004a; AlRashidi et al. 2010, 2011).

The specific aims of our study were to first (i) explore the relationship between the total incubation effort of Kentish Plovers and the nest cover (range: 0 to 100%) in a Mediterranean habitat on the Tunisian coast characterised by mild to warm climate (ground surface temperature doesn't exceed 50°C, HB unpublished data). We predicted that parents nesting in low cover sites will have greater total incubation duration, particularly during the hottest daytime periods, compared to highly covered nests, if overhanging vegetation helps mitigate heat stress on eggs, allowing incubating parents to spend less time on the nest. Alternatively, if temperatures in our study system are not high enough to create thermal constraints, we may not observe a difference in incubation effort across nest cover (i.e., thermal range encountered by nesting parents could be close to the thermoneutral zone, Amat and Masero 2004a). Second, (ii) we examined whether there is a difference in the frequency and average duration of off‐nest periods across nest cover. We expected open nests to have a higher and more fluctuating ambient temperatures (Clauser and McRae 2017), thus we predicted parents of low cover nests would have shorter mean diurnal off‐nest duration (leave the nest unattended for shorter periods to limit egg heat stress), but higher mean off‐nest frequency (important for parental foraging and to avoid parental hyperthermia) and the opposite pattern should be shown for the high covered nests (AlRashidi et al. 2011). Alternatively, if predation risk rather than temperature constrains the incubation behaviour, we predicted that parents of highly covered nests (with limited visibility) would have higher mean off‐nest frequency as parents must leave the nest to check its surrounding and assess threats (Schneider and Griesser 2013). Finally, (iii) we examined the sex specific differences in incubation effort across nest cover. We expected sexes to differ in their incubation behaviour across nest cover. Male Kentish Plovers typically incubate in the evening hours but can also contribute to incubation in hotter parts of the day to assist the female (Amat and Masero 2004a; AlRashidi et al. 2010, 2011). Therefore, we expected males to have a higher incubation contribution at lower cover nests and we predicted this increased male contribution to be observed more in the diurnal periods as the increased temperatures during daylight require males to help females (AlRashidi et al. 2011).

## 2 Methods

### 2.1 Study Site

We studied a wild population of Kentish Plover located at an operating fishpond near Mahres, Tunisia (34°25.616′N, 10°20.851′ E) between April 4 and July 13 in 2022. The fishpond has a surface of 1.5 km^2^ and is characterised by dykes with sparse vegetation and soil scattered with marine shells. It is also adjacent to a coastal saltmarsh, where the tidal activity of the Gulf of Gabes provides diverse food resources for breeding shorebirds. The mean monthly temperatures during the breeding season generally range between 15°C and 30°C (see Supporting Information S1 for more details and Figure S1). Sympatric nesting species include Common Redshanks ( *Tringa totanus* ), Little Terns ( *Sternula albifrons* ) and Common Terns ( *Sterna hirundo* ). Local predators of Kentish Plover nests include the Montagu's Harrier ( *Circus pygargus* ), Common Tern, Red Foxes ( *Vulpes vulpes* ) and Feral Dogs ( *Canis familiaris* ). The site also has potential agents of human disturbance as a result of fish harvesting activities which take place during the nesting season, across different locations within the fishpond.

### 2.2 Field Methods

We searched for nests by scanning for incubating parents or observing nesting behaviour such as the broken wing display (de Framond et al. 2022). Upon detecting a nest, we assigned it a unique identity and estimated clutch age via egg floatation following the protocol described by Székely et al. (2008). We assessed the nest cover percentage (nest cover %) after clutch completion (i.e., following the laying of the final egg) by estimating from 1 m above the nest the proportion of the nest scrape shaded by overhanging vegetation (0%–100%, Lomas et al. 2014; Kwanye et al. 2024). To assess the reliability of the method we used to estimate nest cover in the field, two observers independently scored nest cover from photographs of the nests after fieldwork. Photographs were taken from the same perspective as nest cover was scored in the field. We then compared nest cover estimates obtained in the field with those obtained from photographs using Spearman's rank correlation. The results showed a strong correlation between the two methods and confirmed the reliability of our nest cover estimates (Spearman's rank correlation analysis, observer 1: *ρ* = 0.94, *S* = 132.23, *p* < 0.001 and observer 2: *ρ* = 0.95, *S* = 107.45, *p* < 0.001). We aimed to record at least 24 h of continuous incubation behaviour using nest cameras (Wyze Camera V3, Wyze Labs, Seattle, Washington USA) with night visibility. Cameras were powered by a power bank (Ravpower Xtreme series‐RP‐PB41, capacity: 26800mAh) buried below the ground. We positioned the cameras 40–50 cm from the nests and camouflaged them with materials from the surrounding nest habitat such as shells or dry Mediterranean Tapeweed leaves ( *Posidonia oceanica* ) to minimise disturbance to incubating parents and avoid predator attraction (Figure S2).

We aimed to collect incubation data from nests with clutches aged between 4 and 19 days of incubation, to exclude atypical incubation schedules just after laying or before hatching (Vincze et al. 2013). In total, our dataset included recordings from 24 nests with nest cover of 38.38% ± 41.08% (mean ± SD, range: 0%–100%). For the majority of nests (*N* = 15), the identity of parents was known via unique colour ring combinations, whereas at a subset of nests the parents were unringed (see Supporting Information S1 for details). The sex of parents was determined by sex‐specific plumage characteristics (Argüelles‐Ticó et al. 2016). The clutch size of the nests ranged from two to three eggs.

### 2.3 Video Recordings Analysis

We coded incubation behaviour from video recordings using BORIS software (version 7.13.9, Friard and Gamba 2016). We coded continuous nest camera footage using three primary categories including male care, female care and parental absence. Male care and female care included either direct incubation or egg shading by males or females. Parental absence included those periods where neither parent was incubating or shading.

### 2.4 Data Preparation

For each recording we excluded the initial time period between the camera setup and the resumption of incubation by any parent to avoid including stress related behaviours that may result from camera placement; this period of time varied across nests (0.34 ± 0.35 h, mean ± SD, range: 0.04–1.43 h). We divided the dataset into 12 2‐h time periods representing the time of the day (following AlRashidi et al. 2010 and AlRashidi et al. 2011). To ensure sufficient temporal sampling within each time period, we excluded time periods with less than 1 h (3600 s) of sampling. The dataset included 12 time periods for 20 nests and 11 time periods for 4 nests. All analyses were performed using the reduced dataset that included an average recording length of 23.22 ± 0.76 h (range: 21.47–23.93 h).

### 2.5 Statistical Analysis

We conducted our analyses using two model types: linear models (LMs) and linear mixed‐effects models (LMMs, ‘lmer’ function from the ‘lme4’ package, Bates et al. 2015). We checked the normality assumptions of the response variables graphically prior to analysis. LMs included three explanatory variables: (1) nest cover %; (2) clutch age (days since clutch completion); and (3) Julian date (date of the recording, days since 1st January). LMMs included the same three variables plus (4) time of the day; (5) the interaction between nest cover % and time of day; and (6) the nest identity as a random effect to account for repeated measures within nests. For both model types, model simplification was carried out by sequentially removing individual predictors and assessing their effect on model fit. Parameter significance for LMs was evaluated using *F*‐tests and for LMMs using likelihood ratio tests (LRTs). Main effects were tested after removal of the non‐significant interaction term.

Given that parents were not uniquely colour ringed at nine nests, it is possible that some unringed parents could appear with more than one nest in the dataset, if they nested early in the season and then renested later in the season, leading to non‐independence across recordings. However, this is unlikely because the laying dates and periods of nest activity typically overlap, indicating that most nests are from different individuals. In addition, the majority of the nests without ringed parents occurred towards the end of the season (see Figure S3). However, to ensure this did not affect our results, we excluded the unknown parents' nests and repeated our analyses and the patterns remained qualitatively similar using this reduced dataset (see Figure S4a,b). Thus, throughout we retained these nests in analyses reported in the main text.

### 2.6 Total Incubation

To investigate the nest cover effect on the total incubation, we defined the total incubation as the proportion of time the nest was incubated by either parent (parent either directly incubating or shading the eggs). We performed an LM using the total incubation arcsine square root transformed (to meet the normality of the residuals) as a response variable. In addition, we investigated the effect of our explanatory variables on the variation in total incubation across the time of the day by performing an LMM using the proportion of incubation for each 2‐h interval (arcsine square root transformed) as a response variable. Furthermore, we also investigated the effect of nest cover as a binary explanatory variable (see Supporting Information S1). This approach yielded similar results.

### 2.7 Diurnal Off‐Nest Behaviour

Given that we expected that the highest temperatures will be observed during daylight in our study system (Amat and Masero 2004a), we first restricted our dataset to diurnal hours only (ranging from 06:00 to 18:00). Second, we computed the diurnal mean off‐nest duration, defined as the total duration of off‐nest periods divided by the number of off‐nest periods (i.e., number of parental absences between 06:00 and 18:00). We then performed an LM using the diurnal mean off‐nest duration (log transformed to meet the normality of the residuals) as a response variable. In addition, we computed the diurnal mean off‐nest frequency, defined as the mean number of cases when parents left the nest without incubation per hour within our diurnal time window. We then performed an LM with the diurnal mean off‐nest frequency as a response variable.

### 2.8 Male Share of Incubation

To investigate whether the male share of incubation is related to nest cover, we defined the male share of incubation as the proportion of incubation carried out by the male, computed by dividing the total time of male share (male either directly incubating or shading the eggs) by the total time of care (male and female combined). We then performed an LM using the male share of incubation (arcsine square root transformed) as a response variable. In addition, we investigated the effect of nest cover on the variation in male share of incubation across the time of the day by performing an LMM using the proportion of male share of incubation for each 2‐h interval (arcsine square root transformed) as a response variable. Finally, we tested whether the male share of incubation is significantly higher than 0.5 by using a one‐sample *t*‐test, where the proportion of male share of incubation was arcsine square root transformed.

We performed all the analysis using R version 4.5.2 (R Core Team 2025) and statistical significance was accepted when *p*‐value < 0.05. Throughout the statistical analyses reported here nest cover was a continuous variable, and the low (< 30%) and high (≥ 30%) cover categorical classification (see Supporting Information S1) was used only for visualisation purposes.

## 3 Results

### 3.1 Total Incubation

**FIGURE 1:**
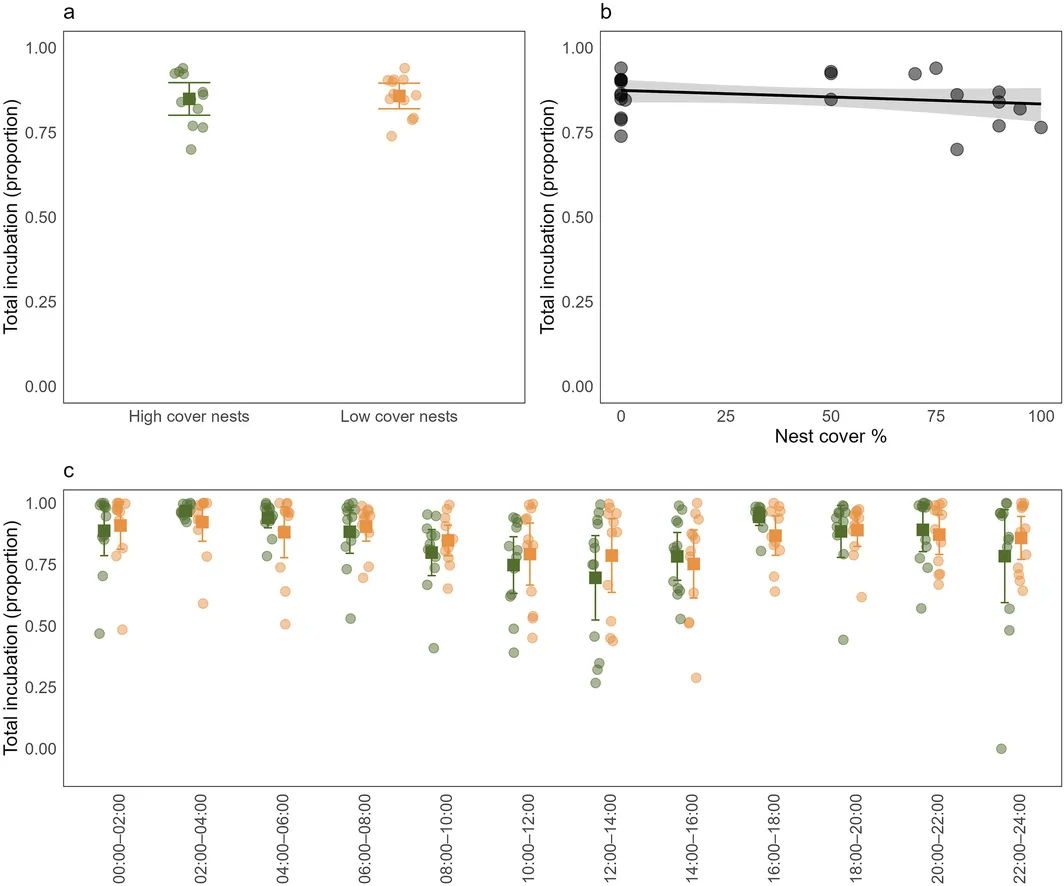
(a) Proportion of total incubation for high (in green) and low (in yellow) nest cover types. (b) Relationship between nest cover and proportion of total incubation. (c) Proportion of total incubation across the time during the day and the nest cover types. Each time interval on the x‐axis represents a 2‐h interval. On panel (a) and (c), the coloured squares represent the mean of total incubation proportion for each cover type (green for high cover, *N* = 12 and yellow for low cover, *N* = 12). The coloured vertical lines show the 95% confidence intervals around each mean estimate. Semi‐transparent coloured points show the raw datapoints for each nest cover type. On panel (b) shaded area shows the 95% confidence interval and the semi‐transparent circles show the raw datapoints (*N* = 24 nests).

Nest cover was not associated with the total incubation across a full day of recording (Table 1, Figure 1a,b). Similarly, clutch age and Julian date were not related to total incubation (Table 1). The total incubation varied throughout the day with a lower proportion of incubation in the afternoon and higher incubation proportion during the morning and the evening (Table 2, Figure 1c). Nest cover, clutch size and Julian date were not associated with the total incubation across a full day (Table 2).

**TABLE 1:** Linear model results assessing incubation behaviour in Kentish Plovers. Degrees of freedom (df) for all *F*‐values and *p*‐values are df = 1.20.

Explanatory variables	Response variable							
	Total incubation		Diurnal mean off‐nest duration		Diurnal mean off‐nest frequency		Male share of incubation	
	*F*	*p*	*F*	*p*	*F*	*p*	*F*	*p*
Nest cover %	1.447	0.242	0.028	0.868	3.821	0.064	0.344	0.563
Clutch age	0.575	0.456	0.037	0.849	0.003	0.955	0.248	0.623
Julian date	0.589	0.451	1.309	0.266	1.499	0.234	1.037	0.320

**TABLE 2:** Linear mixed‐effects model results assessing incubation behaviour in Kentish Plovers across time of the day.

Explanatory variables	Response variable			
	Total incubation		Male share of incubation	
	*χ* ^2^	*p*	*χ* ^2^	*p*
Nest cover % (df = 1)	1.804	0.179	0.29	0.590
Clutch age (df = 1)	0.659	0.417	0.86	0.354
Julian date (df = 1)	0.713	0.398	2.87	0.090
Time of the day (df = 11)	52.135	< 0.001	328.48	< 0.001
Time of the day: nest cover % (df = 11)	10.658	0.472	9.338	0.590

*Note:* Non‐significant interactions were removed before testing the main effects.

### 3.2 Diurnal Off‐Nest Behaviour

**FIGURE 2:**
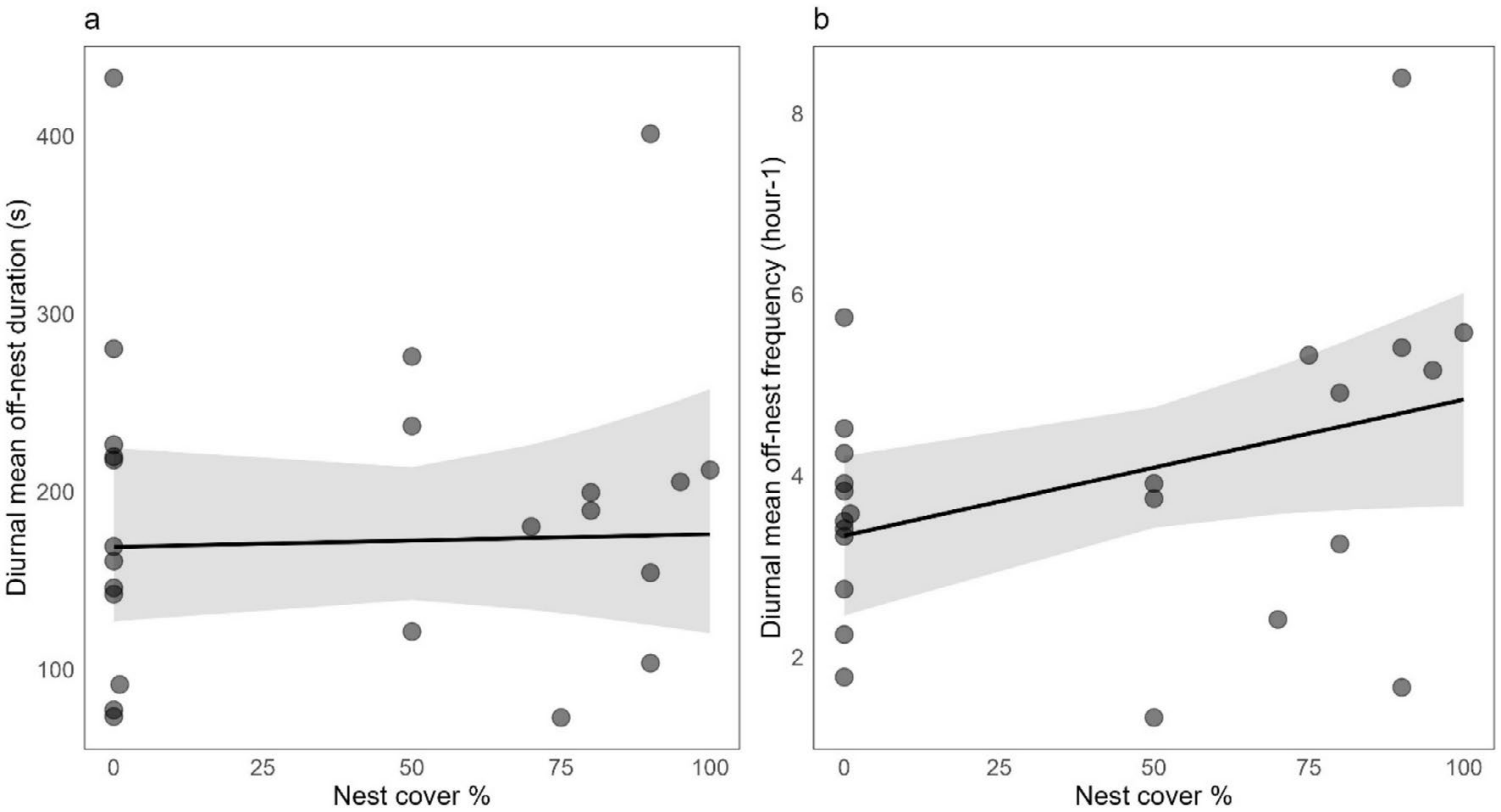
(a) Relationship between nest cover and the mean diurnal off‐nest duration (in seconds). (b) Relationship between nest cover and the diurnal mean off‐nest frequency. Shaded areas show the 95% confidence interval and the semi‐transparent circles show the raw datapoints (*N* = 24 nests).

We found no evidence for a relationship between nest cover and the diurnal mean off‐nest duration (Table 1, Figure 2a). Similarly, we found no evidence that clutch age and Julian date were related to mean diurnal off‐nest duration (Table 1). In addition, nest cover had a borderline non‐significant relationship with diurnal mean off‐nest frequency, with higher nest cover associated with increased off‐nest frequency (Table 1, Figure 2b). There was no evidence that clutch age and Julian date were correlated with the diurnal mean off‐nest frequency (Table 1).

### 3.3 Male Share of Incubation

**FIGURE 3:**
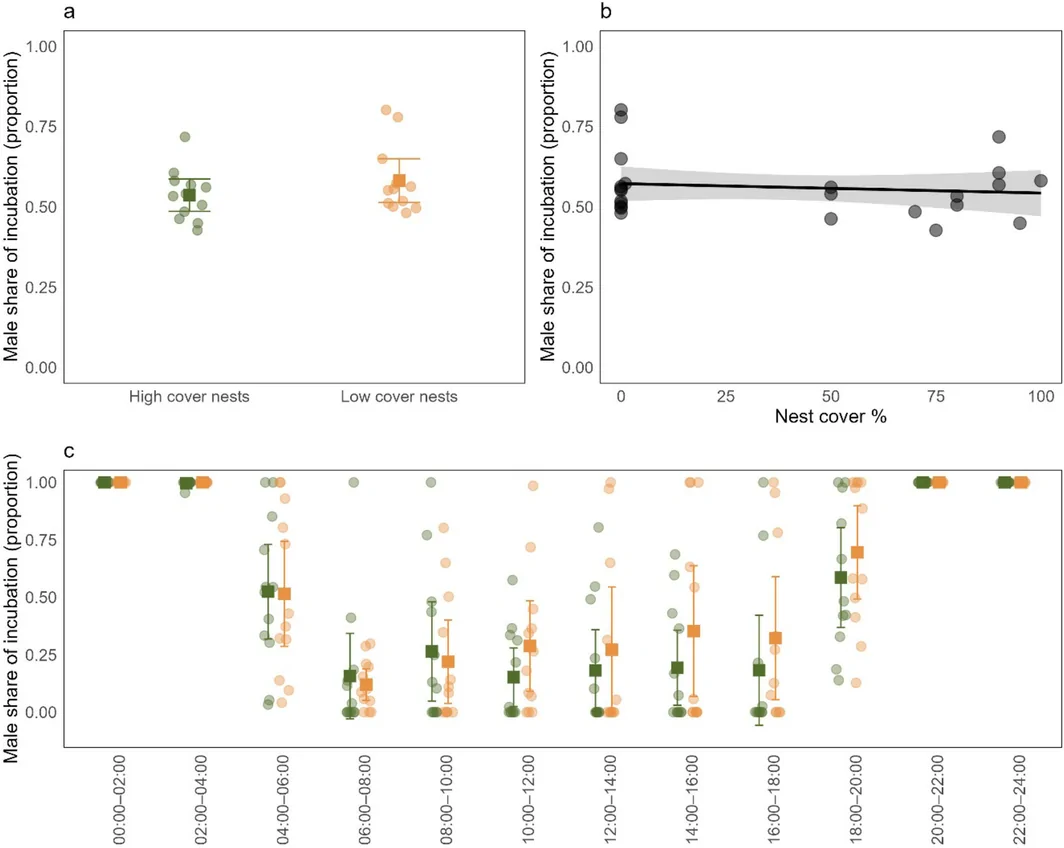
(a) Proportion of male share of incubation for high (in green) and low (in yellow) nest cover types. (b) Relationship between the male share of incubation and the nest cover. (c) Proportion of male share of incubation across the time of the day and nest cover types. Each time interval on the x‐axis represents a 2‐h interval. On panel (a) and (c) the coloured squares represent the mean of male share proportion for each cover type (green for high cover, *N* = 12 and yellow for low cover, *N* = 12). The coloured vertical lines show the 95% confidence intervals around each mean estimate. Semi‐transparent coloured points show the raw datapoints for each nest cover type. On panel (b) shaded area shows the 95% confidence interval and the semi‐transparent circles show the raw datapoints (*N* = 24 nests).

The male share of incubation was not significantly related to nest cover (Table 1, Figure 3a,b). Similarly, clutch age and Julian date were not related to male share of incubation (Table 1). The proportion of the male share of incubation varied throughout the day with a higher care proportion early in the morning and late in the evening (Table 2, Figure 3c). The nest cover was not related to the proportion of male share of incubation across the day (Table 2). The Julian date had a borderline non‐significant relationship with male incubation share, with an increase of males' care proportion as the season progressed (β ± SE = 0.002 ± 0.001, Table 2). In addition, clutch age did not relate to the total proportion of males' care across the day (Table 2). The male share of incubation was significantly higher than 0.5 over the whole day (0.561 ± < 0.001, 95% CI [0.519, 0.603] one sample *t*‐test, *t* (23) = 16.928, *p* < 0.001, Figure 3a).

## 4 Discussion

Our research focused on testing whether the previously reported influence of nest cover on incubation behaviour is general across milder climates. Our study revealed that in our population, nest cover was not associated with the total incubation, off‐nest durations, or the male share of incubation. However, we did identify a potential trend towards a higher off‐nest frequency for nests with a higher nest cover. The incubation effort varied with the circadian rhythm (i.e., the total incubation varied through the daily solar cycle) as parents reduced their incubation effort during the afternoon. Notably, we found a trend towards an increased male share of incubation with the progress of the season and showed that males had higher overall incubation effort compared to females, as evening incubation was conducted almost exclusively by males, and males regularly contributed to incubation throughout daylight hours.

Our study revealed several unexpected patterns that contrast with our initial hypotheses and previous work. Contrary to the prediction that parents of open nests would have greater incubation effort to mitigate heat stress (Amat and Masero 2004a; AlRashidi et al. 2011), we found that the total incubation was not associated with nest cover in our study system. The lack of difference in total incubation across nest cover could be explained by the fact that the ambient temperatures in our study system might have stayed within the Kentish Plovers' thermoneutral zone, making the nest cover less critical than in other more hot environments (Amat and Masero 2004a). Nevertheless, even if thermal conditions did not alter overall incubation durations across nest cover, it is possible that parents respond in more subtle ways (i.e., more thermoregulatory behaviour observed in less covered nests, Amat and Masero 2004a) and future work should interrogate this possibility. In addition, we showed that total incubation varied throughout the full day, consistent with other nesting shorebirds (Xie et al. 2026), such that the lowest total incubation was during afternoon periods. However, we did not reveal any association between the nest cover and the variation in total incubation throughout the full day. This contradicts our assumptions and previous work, as we expected that parents of nests with low cover will not leave the nest unattended particularly in the hottest hours of the day (AlRashidi et al. 2011). Indeed, leaving the nest unattended during the hottest time of the day could lead to embryonic mortality particularly in extremely hot environments, thus parents must remain at the nest to shade the eggs and to avoid eggs overheating risks (AlRashidi et al. 2010). It may be that, in moderately warm habitats, temperatures may remain in a range that is benign to embryos (without overheating risk) and allow incubating parents to leave the nest to forage without harming the eggs (Vleck 1981). Alternatively, lower incubation during hottest hours of the day may occur because parents must prioritise their own homeostasis over the nest incubation to avoid hyperthermia or dehydration risk (Bourne et al. 2021). For example, White‐faced Plover (*Anarhynchus dealbatus*) parents appear to reduce their shift durations close to peak temperatures at midday, potentially as a response to heat stress (Xie et al. 2026). However, previous studies on Kentish Plover in extremely hot habitats showed that parents coordinate their incubation shifts meticulously so that the eggs are consistently shaded by one of the parents while the other parent thermoregulates (AlRashidi et al. 2010, 2011). Another potential explanation for this result is that disturbance agents, lead parents of both high and low cover nests to flee the nest more frequently, decreasing their incubation during midday and afternoon. Human disturbance at our study site is likely much lower than that experienced by Kentish Plovers breeding in other Mediterranean populations characterised by intensive tourism and recreation (e.g., Gómez‐Serrano 2021; Chabani et al. 2026). However, fish harvesting activities may have represented a source of disturbance for incubating parents at our study site. Such disturbance may have led parents regardless of their nest cover to flee their nests more frequently, potentially decreasing parental incubation during the hottest hours of the day, and making differences in incubation behaviour across nest cover cryptic. While our data collection does not allow us to determine the timing and proximity of such disturbance to observed nests, future work should explore how the timing of disturbance may influence overall incubation schedules across differing thermal regimes.

In addition, our study did not reveal any association between nest cover and the off‐nest behaviour (mean off‐nest duration and mean off‐nest frequency), a pattern that also contrasts with our predictions. We expected that parents at exposed nests would have shorter mean diurnal off‐nest duration (leave the nest unattended for shorter periods compared to nests with higher cover), but higher mean off‐nest frequency (more frequent recesses or more changeovers between the parents compared to nests with higher nest cover, Amat and Masero 2004a; AlRashidi et al. 2011, Clauser and McRae 2017). Despite this, the lack of difference in the off‐nest behaviour does not rule out the existence of differences in the extent of parental heat stress. Parents nesting in the open may experience greater heat stress, but instead of altering their off‐nest behaviour, they may adjust how they incubate (e.g., shading the nest, panting, ptiloerection and holding wings) to prevent overheating and maintain the eggs in a tolerable thermal range (Amat and Masero 2004a; Clauser and McRae 2017). In contrast, parents of high nest cover may experience less thermal stress and therefore show heat dissipation behaviours less frequently (Amat and Masero 2004a). While, we did not assess fine‐scale parental heat stress behaviour during incubation in the current study, we suggest future studies should investigate this possibility in combination with nest temperature loggers, to better understand how local nest temperatures may differentially impact overall incubation and parent heat stress. It is also possible that the lack of difference in incubation patterns across nest cover could be linked to predation risk. Although high nest cover may allow parents to remain away from the nest for longer periods, increased nest unattendance is known to increase predation risk, particularly in biparental ground‐nesting birds (Smith et al. 2012; Meyer et al. 2020). In addition, the positive trend where off‐bout frequency increases with nest cover percentages aligns with our predictions and previous work that parents with limited visibility may rely more on auditory stimuli during incubation and respond more strongly to such stimuli, leading to more incubation disruptions as parents may leave the nest to assess their surroundings compared to parents of open nests (Schneider and Griesser 2013; Basso and Richner 2015).

Furthermore, contrary to our expectations we did not reveal any association between the male share of incubation and nest cover. Similarly, we did not find any correlation between the male share of incubation across the day and the nest cover. While male Kentish Plovers typically incubate during the night (Fraga and Amat 1996; Kosztolányi and Székely 2002), their participation in incubation during the daytime is known to be a response to high temperatures (males cooperate more during daytime incubation under intense heat stress conditions, AlRashidi et al. 2010, 2011). Our study revealed that males contributed to daylight incubation, and although there was considerable variation between individuals (Figure 3c), the mean male share during daylight hours typically remained well below 50%. This contrasts with populations nesting in extremely hot environments where males notably increase their midday incubation share (AlRashidi et al. 2010; AlRashidi et al. 2011). This further suggests that diurnal temperatures in our study system (even during the hottest hours of the day) were within an acceptable thermal range for female parents, thus males were not required to increase their incubation effort (Amat and Masero 2004a; AlRashidi et al. 2011; Vincze et al. 2017). Despite this, we did identify a trend towards an increased male share of incubation later in the season, which may suggest males can scale up their share of incubation at higher temperatures, as monthly average temperatures increase throughout the season in our study site (Figure S1, Amat and Masero 2004a; AlRashidi et al. 2011).

In summary, our study indicates that nest cover is not a consistently strong modulator of incubation schedules in a population of Kentish Plovers, suggesting its effect is not general and it likely varies with local environmental conditions. While we acknowledge that our relatively small sample size may have limited the statistical power, and that non‐significant results may not imply the lack of an effect, our results were able to establish the key expected patterns of incubation, including strong sex differences in incubation timing throughout the day. Our results contrast with observations from extremely hot habitats, suggesting that in our population, ambient temperatures may remain within the thermoneutral zone of the species. Further work is needed to establish whether Kentish Plovers parents in different populations differ in how they behaviourally regulate heat stress, for example through thermoregulatory behaviours rather than altering incubation duration (Amat and Masero 2004a). We speculate that the thermal buffering from vegetation may be less critical than anticipated in some populations, and future work should simultaneously investigate how nest cover may influence egg concealment from aerial predators, shelter incubating parents from wind and rare but potentially impactful heavy rain events (Kim and Monaghan 2005; Gómez‐Serrano and López‐López 2014; Josiah and Downs 2023).

In conclusion, we did not find evidence that nest cover shapes incubation schedules in our studied population of Kentish Plover nesting in less extreme thermal environments. However, the potential role of nest cover in shaping incubation behaviour under different climatic conditions remains an important question. We encourage further research on the incubation behaviour of ground‐nesting biparental birds across a broad climatic gradient, by comparing populations from cold to extremely hot environments, as well as studying the heat stress behaviours employed by incubating parents across nest covers. Such work may help identify differences in the primary function of nest cover across differing environmental conditions, and the specific thermal thresholds at which nest cover transitions to a critical requirement for ensuring an optimal thermal range for incubating parents and embryonic development.

## Author Contributions

**Hela Boughdiri:** conceptualization (equal), data curation (lead), formal analysis (lead), funding acquisition (equal), investigation (lead), methodology (lead), writing – original draft (lead). **Grant C. McDonald:** conceptualization (equal), data curation (supporting), formal analysis (supporting), funding acquisition (equal), investigation (supporting), methodology (supporting), project administration (lead), supervision (lead), validation (lead). **Tamás Székely:** conceptualization (equal), funding acquisition (equal), investigation (supporting), methodology (supporting), project administration (equal), supervision (equal), validation (equal). **András Kosztolányi:** conceptualization (equal), data curation (supporting), formal analysis (supporting), funding acquisition (equal), investigation (supporting), methodology (supporting), project administration (lead), supervision (lead), validation (lead).

## Funding

This work was supported by the National Research, Development and Innovation Office, Hungary to Grant C. McDonald (FK134741, ADVANCED 153335), András Kosztolányi (NN 125642, ANN 143995) and Tamás Székely (ÉLVONAL KKP 126949) and the Tempus Public Foundation to Hela Boughdiri (SHE‐090534‐004/2022).

## Ethics Statement

The research on Kentish Plovers in Tunisia was approved by the General Directorate of Forests, Tunisia (Research Permit No. 1191, issued on 28 March 2022). Camera recordings were installed in a non‐invasive manner to minimise disturbance to the incubating parents.

## Conflicts of Interest

The authors declare no conflicts of interest.

## Supporting information


**Data S1:** ece374225‐sup‐0001‐DataS1.zip.

## Data Availability

The data for this study will be made publicly available upon acceptance (https://doi.org/10.5281/zenodo.19555730).
