# Supplementary material for: No Evidence That Nest Cover Shapes Incubation Schedules in a Mediterranean Shorebird Population
Source: Ecol Evol. 2026 Aug 20;16(8):e74225. doi: 10.1002/ece3.74225 (PMC13492183; doi:10.1002/ece3.74225)
Supplement: Supplementary file 1 [file ECE3-16-e74225-s001.zip › Boughdiri et al_SI_Ecology_and_Evolution_1AUGUST.docx]

**No evidence that nest cover shapes incubation schedules in a Mediterranean shorebird population**

Hela Boughdiri ^1^, Grant C. McDonald ^2^, Tamás Székely ^1,3^, András Kosztolányi ^2^

1 Department of Evolutionary Zoology and Human Biology, University of Debrecen, Debrecen, Hungary

2 Department of Zoology, University of Veterinary Medicine Budapest, Budapest, Hungary

3 Milner Centre for Evolution, Department of Life Sciences, University of Bath, Bath, UK

First corresponding author: Hela Boughdiri, email: hela1994@mailbox.unideb.hu / hela.boughdiri@gmail.com

Second corresponding author: András Kosztolányi, Kosztolanyi.Andras@univet.hu

ORCID of the authors:

Hela Boughdiri: 0000-0001-5508-1619

Grant C. McDonald: 0000-0002-2864-9608

Tamás Székely: 0000-0003-2093-0056

András Kosztolányi: 0000-0002-9109-5871

**Supplementary information**

**Climate model showing the increase in temperature across the breeding season**

We obtained climate data for our study site (Oued Maltine fishpond) from the CRU dataset, version 4.09 (Harris et al. 2020). We analysed the monthly average daily mean temperature (°C) from March to July using a Gaussian linear model with month as predictor. The mean temperature increased significantly over the breeding season (March-July; month effect: F (1, 17) = 235.7, *p* <0.001, Fig. S1).

**Total incubation duration across different nest cover types**

To investigate the variation of total incubation between high and low cover nests, we performed the same Gaussian linear model (LM) but we replaced the continuous nest cover percentage with a categorical variable, classifying nests as either high cover (≥ 30 %) or low cover (< 30 %). This threshold was defined based on field observations because nests with 30% or more cover typically conceal at least one third of the clutch and the parent (for similar classification see Colombelli‐Négrel and Kleindorfer 2009). The results of our model showed no significant effect of nest cover type on the total incubation (LM, F (1, 20) = 0.318, *p* = 0.578). Similarly, the Julian date and the clutch age did not show any association to the total incubation (Julian date: LM, F (1, 20) = 0.496, *p* = 0.489); clutch age: LM, F (1, 20) = 0.514, *p* = 0.481)

**Supplementary figures**

**Fig. S1** Plot showing the monthly average daily mean temperature (degrees Celsius) for the region of our study (Mahres fishpond) during the year of 2022, grey area highlights the study period.

**Fig. S2** Picture of the camera recording deployment in the field. Red arrow shows the camera and red circle shows the Kentish Plover nest under overhanging vegetation

**Fig. S3** Temporal distribution of nests included in the study. Black bars represent nests of uniquely ringed Kentish Plover pairs (N = 15), and pink bars represent nests of plovers with unknown identity (N = 9). For each nest, the left end of the bar indicates the Julian clutch completion date, and the right end indicates the last Julian date when the nest was active

**Fig. S4 a** Proportion of total incubation for high (in green) and low (in yellow) nest cover types from uniquely ringed parents only. The transparent coloured circles show the raw datapoints (high cover nests: N= 7), and low cover nests: N=8). The coloured squares represent the mean proportion of incubation for each cover type. The coloured vertical lines show the 95% confidence intervals around each mean estimate for each nest cover type. **b** Relationship between nest cover % and the proportion of total incubation. Shaded area shows the 95% confidence interval and the semi-transparent circles show the raw datapoints (N= 15 nests)

**References**

Bates D, Maechler M, Bolker B, Walker S (2015) Fitting linear mixed-effects models using lme4. Journal of Statistical Software 67(1):1-48. doi:10.18637/jss.v067.i01

Colombelli‐Négrel D, Kleindorfer S (2009) Nest height, nest concealment, and predator type predict nest predation in superb fairy‐wrens (*Malurus cyaneus*). Ecological Research 24(4):921-928. https://doi.org/10.1007/s11284-008-0569-y

Harris I, Osborn TJ, Jones P, Lister D (2020) Version 4 of the CRU TS monthly high-resolution gridded multivariate climate dataset. Scientific data 7(1):109. doi:10.1038/s41597-020-0453-3
